# Assessing the Accuracy and Comprehensiveness of ChatGPT in Offering Clinical Guidance for Atopic Dermatitis and Acne Vulgaris

**DOI:** 10.2196/50409

**Published:** 2023-11-14

**Authors:** Nehal Lakdawala, Leelakrishna Channa, Christian Gronbeck, Nikita Lakdawala, Gillian Weston, Brett Sloan, Hao Feng

**Affiliations:** 1 University of Connecticut School of Medicine Farmington, CT United States; 2 Department of Dermatology University of Connecticut Health Center Farmington, CT United States; 3 The Ronald O. Perelman Department of Dermatology New York University New York, NY United States

**Keywords:** ChatGPT, artificial intelligence, dermatology, clinical guidance, counseling, atopic dermatitis, acne vulgaris, skin, acne, dermatitis, NLP, natural language processing, dermatologic, dermatological, recommendation, recommendations, guidance, advise, counsel, response, responses, chatbot, chatbots, conversational agent, conversational agents, answer, answers, computer generated, automated

## Introduction

When facing barriers to timely and accessible in-person dermatologic care, patients may look for guidance on skin health from alternative sources like artificial intelligence platforms. ChatGPT, a language processing tool trained on web-based articles, books, and websites, has the potential to enhance information accessibility [1,2]. However, literature assessing its dermatologic utility is scant [3]. This study characterizes the accuracy and comprehensiveness of ChatGPT in responding to common patient questions for acne vulgaris and atopic dermatitis (AD).

## Methods

### Overview

For each condition (acne vulgaris and AD), 32 potential patient questions were created based on American Academy of Dermatology (AAD) guidelines and web-based resource centers [4,5]. To the extent possible, questions were generated using explicit wording from the guideline “clinical questions” and resource center bullet points, and were approved by consensus from board-certified dermatologists. The questions aimed to address various disease aspects and incorporate lay terminology (eg, “blackheads”; Textbox 1 and Multimedia Appendix 1). Each question was presented to ChatGPT-3.5, and the responses were independently graded for quality by board-certified dermatologists.

**Sample of ChatGPT input questions for atopic dermatitis and acne vulgaris. For the comprehensive list of questions, please see the corresponding Appendix.**

**Natural history**

- Atopic dermatitis
  What causes eczema?
  At what age does childhood atopic dermatitis usually start?
  What can trigger flares of atopic dermatitis?
- Acne vulgaris
  What causes acne?
  What is the role of diet in acne vulgaris?
  Why do adults get acne?

**Symptoms and differential diagnosis**

- Atopic dermatitis
  What other conditions are associated with atopic dermatitis?
  Can acne cause scars?
  What are the signs and symptoms of atopic dermatitis in children?
- Acne vulgaris
  How is acne different from rosacea?
  What signs suggest acne is related to polycystic ovarian syndrome?
  How is acne different from hidradenitis suppurativa?

**Treatment and management**

- Atopic dermatitis
  What is the efficacy of topical corticosteroids for the treatment of atopic dermatitis?
  What is the efficacy of phototherapy for the treatment of atopic dermatitis?
  What environmental modifications around the house can be implemented to improve atopic dermatitis?
- Acne vulgaris
  What is the efficacy of benzoyl peroxide in the treatment of acne vulgaris?
  What are the potential side effects of isotretinoin in the treatment of acne vulgaris?
  What topical agents can be combined in the treatment of acne vulgaris?

### Ethical Considerations

This study used publicly available web-based data sets; institutional review board approval was not required at the University of Connecticut Health Center.

## Results

Across both diseases, 78% (50/64) of ChatGPT responses were correct but inadequate (score ≤2), with 45% (29/64) of answers being fully comprehensive (score 1). No responses were completely inaccurate (score 4). For AD and acne specifically, 88% (28/32) and 66% (21 of 32) of responses were correct but inadequate (score ≤2), and 53% (17/32) and 34% (11/32) were fully comprehensive (score 1), respectively. This *broadly* indicates acceptable performance of ChatGPT across both conditions. However, whereas ≥75% of responses for AD *within each category* (including natural history, symptoms and differential diagnosis, and treatment and management) were correct (score ≤2), the accuracy of responses for acne treatment/management (score ≤2) specifically was relatively low (9/16, 56%), suggesting a deficit in ChatGPT in advising treatment recommendations for this condition (Table 1). The interrater reliability, as measured by the weighted Cohen κ coefficient, was 0.44, indicating moderate agreement among the board-certified dermatologists in our study.

**Table 1:** Evaluation of ChatGPT output responses for atopic dermatitis and acne vulgaris.^a^

			Atopic dermatitis (n=8), n (%)		Acne vulgaris (n=8), n (%)		Both conditions (n=16), n (%)
**Natural history**							
	1. Comprehensive	4 (50)		5 (63)		9 (56)	
	2. Correct but inadequate	3 (38)		1 (13)		4 (25)	
	3. Mixed with correct and incorrect/outdated data	1 (13)		2 (25)		3 (19)	
	4. Completely incorrect	0 (0)		0 (0)		0 (0)	
**Symptoms and differential**							
	1. Comprehensive	5 (63)		2 (25)		8 (44)	
	2. Correct but inadequate	3 (38)		4 (50)		7 (44)	
	3. Mixed with correct and incorrect/outdated data	0 (0)		2 (25)		2 (13)	
	4. Completely incorrect	0 (0)		0 (0)		0 (0)	
**Treatment and management^b^**							
	1. Comprehensive	8 (50)		4 (25)		12 (38)	
	2. Correct but inadequate	5 (31)		5 (31)		10 (31)	
	3. Mixed with correct and incorrect/outdated data	3 (19)		7 (44)		10 (31)	
	4. Completely incorrect	0 (0)		0 (0)		0 (0)	

^a^The table demonstrates scores for ChatGPT responses for atopic dermatitis and acne vulgaris. Each question was presented to ChatGPT-3.5 (queries entered on February 27, 2023, using the February 9, 2023, release version), and response quality was graded independently by two board-certified dermatologists (authors GW and NL) using a scale previously referenced in the literature: (1) comprehensive, (2) correct but inadequate, (3) mixed with correct and incorrect/outdated data, (4) completely incorrect [1]. If reviewers agreed, their score was used. Discrepancies in scoring of ≥1 point (29/64, 45% of responses) were evaluated independently by a third board-certified academic dermatologist (author BS) with over 25 years of clinical experience, and the majority score was used; if there was no majority, the third reviewer score was prioritized. See Multimedia Appendix 2 for a sample of ChatGPT output responses, associated scores, and commentary on response accuracy and comprehensiveness.

^b^For the treatment and management category, 16 responses were evaluated for atopic dermatitis and acne vulgaris each, for a total of 32 responses.

## Discussion

In the realm of dermatological care, platforms like ChatGPT can serve as supplementary educational tools, enhancing information accessibility for patients with conditions such as acne vulgaris or AD. However, the reviewers found that the accuracy and comprehensiveness were lower for questions on acne treatment as compared to AD, occasionally omitting information on treatment effectiveness, lacking guidance on treatment expectations, and failing to provide age-appropriate and patient-specific recommendations. For example, while ChatGPT accurately described the mechanism of tetracyclines in treating acne vulgaris, it did not identify patients with moderate inflammatory acne who are typically candidates for this medication. Similarly, it failed to recognize more recent randomized controlled trials supporting the use of spironolactone in female patients with hormonal acne and stated that it is not a first-line treatment. Acne treatment is nuanced and expanding, and includes a plethora of topical and oral treatments that depend on a patient’s age, gender, severity, skin tone, and preferences, likely minimizing ChatGPT’s ability to provide accurate and patient-specific guidance. While responses were often conversational in tone, they sometimes incorporated advanced terminology that may be confusing for patients (eg, antiandrogen or meta-analysis; Multimedia Appendix 2).

These findings underscore the ethical dilemma surrounding ChatGPT and other artificial intelligence platforms—specifically, whether the benefit of more accessible dermatologic information outweighs the risk of harm from potentially confusing or incomplete information [3]. The findings encourage dermatologists to assess patients’ baseline understanding and inquire about the educational resources used. In addition to targeted counseling, providing patients with educational handouts or links to AAD patient resource centers may be prudent to ensure access to comprehensive, patient-targeted, up-to-date information.

This assessment has limitations. There is inherent variability in ChatGPT’s responses; however, through our extensive interactions with the software, we did not find meaningful differences in the answers’ content when we presented the same question several times. Additionally, our findings’ reliability and applicability are contingent upon the type of generative AI models used and the specific phrasing of questions. These findings also do not apply to queries on all skin conditions. Moreover, this research used the popular ChatGPT-3.5 model; subsequent models with more advanced training may yield varying outcomes.

Nonetheless, these findings provide an initial characterization of the accuracy and comprehensiveness of ChatGPT in two common skin conditions. Additional studies are required to further understand the clinical benefits and risks of artificial intelligence in patient-initiated dermatologic education. Our findings are time sensitive. As AI models like ChatGPT evolve and guidelines update, the performance and relevance of our results with ChatGPT-3.5 may change. Continuous reassessments are crucial for maintaining accuracy and relevance.

## Appendix

Multimedia Appendix 1 ChatGPT input questions and associated scores for atopic dermatitis and acne vulgaris.

Multimedia Appendix 2 Sample ChatGPT output responses for atopic dermatitis and acne vulgaris and associated physician scores and author commentary.

## Abbreviations

AAD: American Academy of Dermatology
AD: atopic dermatitis
