# Supplementary material for: Assessing the Accuracy and Comprehensiveness of ChatGPT in Offering Clinical Guidance for Atopic Dermatitis and Acne Vulgaris
Source: JMIR Dermatol. 2023 Nov 14;6:e50409. doi: 10.2196/50409 (PMC10685272; doi:10.2196/50409)
Supplement: Multimedia Appendix 1 [file derma_v6i1e50409_app1.docx]

**Appendix 1.** ChatGPT input questions and associated scores for atopic dermatitis and acne vulgaris

| **Acne Vulgaris (Score)** | **Atopic Dermatitis (Score)** |
| --- | --- |
| *Natural History* | |
| How is acne graded and classified? (1) | What causes eczema? (2) |
| What is the role of endocrine testing in evaluating patients with acne vulgaris? (1) | What risk factors are associated with atopic dermatitis? (2) |
| What is the role of diet in acne vulgaris? (2) | How is the severity of atopic dermatitis assessed? (2) |
| Why does acne develop in the teenage years? (3) | What can trigger flares of atopic dermatitis? (3) |
| Why do adults get acne? (1) | Are patients of certain racial or ethnic groups more likely to get atopic dermatitis? (1) |
| Can stress cause acne? (1) | What are the most valid and reliable measures for diagnosing atopic dermatitis? (1) |
| Can nicotine cause acne? (1) | At what age does childhood atopic dermatitis usually start? (1) |
| What causes acne? (3) | Do children outgrow eczema? (1) |
| *Symptoms and Differential* | |
| What are blackheads? (2) | What are the signs and symptoms of atopic dermatitis in infants? (2) |
| Can acne cause scars? (2) | What are the signs and symptoms of atopic dermatitis in children? (2) |
| What other diseases can be associated with acne? (3) | What are the signs and symptoms of atopic dermatitis in adults? (1) |
| Can acne affect your mental health? (1) | What other conditions are associated with atopic dermatitis? (1) |
| How is acne different from rosacea? (2) | Can atopic dermatitis affect your mental health? (1) |
| What signs suggest acne is related to polycystic ovarian syndrome? (1) | How is atopic dermatitis different from psoriasis? (2) |
| How is acne different from hidradenitis suppurativa? (2) | How is atopic dermatitis different from dry skin? (1) |
| How is acne different from perioral dermatitis? (3) | How is atopic dermatitis different from a skin infection? (1) |
| *Treatment and Management* | |
| What is the efficacy of retinoids in the treatment of acne vulgaris? (1) | What is the efficacy of topical corticosteroids for the treatment of atopic dermatitis? (1) |
| What are the potential side effects of retinoids in the treatment of acne vulgaris? (3) | What is the optimal dose and frequency of topical corticosteroids for the treatment of atopic dermatitis? (1) |
| What is the efficacy of benzoyl peroxide in the treatment of acne vulgaris? (2) | What are the adverse effects of topical corticosteroids for the treatment of atopic dermatitis? (3) |
| What is the efficacy of azelaic acid in the treatment of acne vulgaris? (2) | What is the efficacy of topical calcineurin inhibitors for the treatment of atopic dermatitis? (1) |
| What is the efficacy of tetracyclines in the treatment of acne vulgaris? (3) | What is the efficacy of topical Janus kinase inhibitors for the treatment of atopic dermatitis? (3) |
| What is the efficacy of contraceptive agents in the treatment of acne vulgaris? (3) | In what situations are topical antimicrobials prescribed for atopic dermatitis? (3) |
| What is the efficacy of spironolactone in the treatment of acne vulgaris? (3) | What is the efficacy of prednisone in the treatment of atopic dermatitis? (2) |
| What is the effiacy of isotretinoin in the treatment of acne vulgaris? (2) | What is the optimal dose and frequency of oral prednisone for the treatment of atopic dermatitis? (2) |
| What are the potential side effects of isotretinoin in the treatment of acne vulgaris? (2) | What is the efficacy of systemic antihistamines for the treatment of atopic dermatitis? (1) |
| What topical agents can be combined in the treatment of acne vulgaris? (3) | What is the efficacy of phototherapy for the treatment of atopic dermatitis? (2) |
| Is it ok to wear makeup with acne? (1) | What is the efficacy of dupilumab in the treatment of atopic dermatitis? (1) |
| How often should I wash my face with acne? (1) | What is the efficacy of upadacitinib in the treatment of atopic dermatitis? (1) |
| Can I pop my own acne pimples? (1) | What is the utility of screening for allergens in patients with atopic dermatitis? (2) |
| How is acne treated in skin of color? (2) | What is the efficacy of dietary interventions or dietary restriction in the treatment of atopic dermatitis? (1) |
| How do I treat dark spots related to acne? (3) | What environmental modifications around the house can be implemented to improve atopic dermatitis? (2) |
| What can be used to treat acne on the back? (3) | When and how should moisturizers be applied for atopic dermatitis? (1) |

This table denotes the comprehensive list of 64 patient questions (32 for each condition) used as input for ChatGPT for acne vulgaris and atopic dermatitis, along with the final score after review by independent board-certified dermatologists. The responses were evaluated on the following 4-point scale: 1. Comprehensive, 2. Correct but inadequate, 3. Mixed with correct and incorrect/outdated data, 4. Completely incorrect.
