# Supplementary material for: Assessing the Accuracy and Comprehensiveness of ChatGPT in Offering Clinical Guidance for Atopic Dermatitis and Acne Vulgaris
Source: JMIR Dermatol. 2023 Nov 14;6:e50409. doi: 10.2196/50409 (PMC10685272; doi:10.2196/50409)
Supplement: Multimedia Appendix 2 [file derma_v6i1e50409_app2.docx]

**Appendix 2.** Sample ChatGPT output responses for atopic dermatitis and acne vulgaris and associated physician scores and author commentary

| What causes acne? | Acne is a common skin condition that occurs when hair follicles become clogged with oil and dead skin cells. The exact cause of acne is not fully understood, but several factors can contribute to its development. Some of the most common causes of acne include: |
| --- | --- |
| (Score: 3) |  |
|  | Hormonal changes: Hormonal changes during puberty, menstrual cycles, and pregnancy can cause the skin to produce more oil, leading to acne. |
|  | Excess oil production: Excessive oil production can clog hair follicles and lead to the formation of acne. |
|  | Bacteria: Bacteria on the skin, particularly Propionibacterium acnes, can also contribute to the development of acne. |
|  | Genetics: Acne can run in families, and genetic factors may play a role in its development. |
|  | Environmental factors: Exposure to certain environmental factors, such as pollution and humidity, can also contribute to acne. |
|  | Diet: Some studies suggest that certain foods, such as dairy products and high-glycemic-index foods, may worsen acne in some people. |
|  | Medications: Some medications, such as corticosteroids and hormones, can also cause acne. |
|  | Overall, acne is a multifactorial condition, and its development often involves a combination of several of these factors. |
|  | *Author Commentary:* While ChatGPT is generally correct, there are a few generalizations and important omissions in this response. The role of diet can be controversial. The link between high-glycemic foods and acne vulgaris is well-established, although dairy (in the form of milk) consumption may only contribute to acne in certain populations. There is also no evidence that yogurt or cheese can worsen acne. While the list of medications provided is accurate, there are several additional medications that have a significant known association with acne vulgaris (e.g., lithium, anti-seizure medications) that are not mentioned. |
| What signs suggest acne is related to polycystic ovarian syndrome? | Acne is a common skin condition that can have a variety of causes, including hormonal imbalances. Polycystic ovarian syndrome (PCOS) is a hormonal disorder that affects women of reproductive age and is known to cause hormonal imbalances. |
| (Score: 1) |  |
|  | Some signs that suggest acne may be related to PCOS include: |
|  | Acne that is persistent and does not respond well to conventional treatments, such as over-the-counter medications or topical creams. |
|  | Acne that is accompanied by other symptoms of PCOS, such as irregular periods, weight gain, and excess facial and body hair. |
|  | Acne that appears on the jawline, chin, and cheeks, which are common areas affected by hormonal imbalances. |
|  | Acne that worsens during menstrual cycles or times of hormonal fluctuations, such as during pregnancy or menopause. |
|  | If you are experiencing persistent acne and any of the above symptoms, it is recommended that you see a healthcare provider for an evaluation. They can help determine if your acne is related to PCOS and provide appropriate treatment options. |
| What is the efficacy of spironolactone in the treatment of acne vulgaris? (Score: 3) | Spironolactone is an anti-androgen medication that is sometimes prescribed off-label for the treatment of acne vulgaris, particularly in women with hormonal acne. |
|  | There is evidence to suggest that spironolactone can be effective in reducing the severity of acne. A number of small-scale studies and case reports have found that spironolactone can improve acne in women by reducing sebum production and blocking the effects of androgen hormones on the skin. However, more large-scale randomized controlled trials are needed to establish its safety and efficacy in the treatment of acne. |
|  | It is important to note that spironolactone is not typically used as a first-line treatment for acne vulgaris, and should only be used under the supervision of a healthcare professional. It can have side effects and may not be appropriate for everyone. Other treatments, such as topical and oral antibiotics, topical retinoids, and oral contraceptives, may be more appropriate for some patients. |
|  | *Author Commentary:* While ChatGPT accurately outlines the mechanism of spironolactone for acne vulgaris, it is incorrect that there are only small-scale studies and case reports demonstrating its efficacy. There are more recent randomized-controlled trials that have demonstrated its efficacy in treating acne vulgaris in female patients and it often is used as a first-line treatment in the appropriate female patient with hormonal acne. Additionally, it fails to provide any efficacy estimates from the literature. |
| What is the efficacy of tetracyclines in the treatment of acne vulgaris? (Score: 3) | Tetracyclines are a group of antibiotics that are commonly used to treat acne vulgaris, a common skin condition that affects many people, especially adolescents. Tetracyclines work by inhibiting the growth of Propionibacterium acnes, a bacteria that is involved in the development of acne. |
|  | Several studies have demonstrated the efficacy of tetracyclines in the treatment of acne vulgaris. In general, tetracyclines are effective in reducing the severity of acne lesions, such as papules, pustules, and nodules. They can also help prevent the formation of new acne lesions. |
|  | Tetracyclines are usually prescribed as a short-term treatment for acne, typically for several weeks to a few months. Long-term use of tetracyclines is generally not recommended due to the risk of developing antibiotic resistance. |
|  | It is important to note that tetracyclines are not suitable for everyone, and their use can be associated with certain side effects, such as gastrointestinal disturbances, photosensitivity, and tooth discoloration. Therefore, it is important to consult with a healthcare provider before starting treatment with tetracyclines or any other medication for acne vulgaris. |
|  | *Author Commentary:* While ChatGPT correctly outlines the mechanism of tetracycline medications in treating acne vulgaris, there are several important omissions. Importantly, the efficacy descriptions are vague. Additionally, low-dose tetracyclines (e.g., doxycycline) are increasingly utilized at sub-antimicrobial doses for the treatment of acne vulgaris, with similar effiacy to higher doses and with greater long-term safety (e.g., more than a few months). Finally, while ChatGPT accurately describes the mechanism of tetracyclines in treating acne vulgaris, it does not identify the types of patients who are typically candidates for this medication (those with moderate inflammatory acne). |
| What causes eczema? (Score: 2) | Eczema, also known as atopic dermatitis, is a chronic skin condition that can cause redness, dryness, itching, and rash on various parts of the body. The exact cause of eczema is not fully understood, but it is believed to be a combination of genetic and environmental factors. |
|  |  |
|  | Here are some of the factors that can contribute to eczema: |
|  | Genetics: Eczema tends to run in families, suggesting that there may be a genetic component to the condition. |
|  | Abnormal immune system function: People with eczema tend to have an overactive immune system, which can lead to inflammation and skin irritation. |
|  | Environmental factors: Exposure to certain environmental factors, such as harsh soaps, detergents, and fabrics, can trigger eczema symptoms. |
|  | Allergies: Allergies to food, pollen, dust mites, and other substances can contribute to eczema. |
|  | Stress: Stress can weaken the immune system and trigger eczema symptoms in some people. |
|  | Skin barrier dysfunction: People with eczema tend to have a weakened skin barrier, which allows irritants and allergens to penetrate the skin more easily.  It's important to note that eczema can vary widely from person to person, and what triggers symptoms in one person may not affect another. |
|  |  |
| What other conditions are associated with atopic dermatitis? | Atopic dermatitis, also known as eczema, is a chronic inflammatory skin condition that affects millions of people worldwide. Here are some other conditions that are commonly associated with atopic dermatitis: |
| (Score: 1) |  |
|  | Asthma: Atopic dermatitis and asthma often occur together, especially in children. In fact, up to 80% of children with atopic dermatitis also develop asthma. |
|  | Hay fever: Also known as allergic rhinitis, hay fever is another condition that often coexists with atopic dermatitis. It is characterized by sneezing, runny nose, and itchy eyes. |
|  | Food allergies: People with atopic dermatitis may also have food allergies, particularly to common allergenic foods such as milk, eggs, peanuts, and soy. |
|  | Contact dermatitis: This type of dermatitis occurs when the skin comes into contact with an irritant or allergen, leading to a rash or other skin symptoms. People with atopic dermatitis may be more prone to developing contact dermatitis. |
|  | Stress and anxiety: Emotional stress and anxiety can exacerbate atopic dermatitis symptoms, and some people with atopic dermatitis may also experience anxiety or depression. |
|  | Other skin infections: People with atopic dermatitis may be more prone to developing other skin infections such as impetigo, a bacterial skin infection that causes red sores on the face and other parts of the body. |
|  | It's important to note that not everyone with atopic dermatitis will develop these associated conditions, and having one or more of these conditions does not necessarily mean that a person has atopic dermatitis. It's always best to consult with a healthcare professional for an accurate diagnosis and appropriate treatment. |
| What is the efficacy of topical corticosteroids for the treatment of atopic dermatitis? (Score: 1) | Topical corticosteroids are a commonly used treatment for atopic dermatitis, also known as eczema. These medications work by reducing inflammation and itching in the skin. |
|  | The efficacy of topical corticosteroids for treating atopic dermatitis varies depending on the severity of the condition and the specific corticosteroid used. In general, higher potency corticosteroids tend to be more effective, but they also come with a greater risk of side effects. |
|  | A meta-analysis of randomized controlled trials on the use of topical corticosteroids for atopic dermatitis found that they were significantly more effective than placebo at reducing symptoms such as itching, redness, and scaling. The study also found that the benefits of treatment with topical corticosteroids were seen in both adults and children, and across different severities of the disease. |
|  | However, prolonged and frequent use of topical corticosteroids can lead to skin thinning, discoloration, and other side effects. Therefore, it is important to use these medications as directed by a healthcare provider and to avoid overuse or misuse. In some cases, alternative treatments such as moisturizers, phototherapy, or immunosuppressive medications may be recommended for long-term management of atopic dermatitis. |
|  | Contact dermatitis: This type of dermatitis occurs when the skin comes into contact with an irritant or allergen, leading to a rash or other skin symptoms. People with atopic dermatitis may be more prone to developing contact dermatitis. |
|  | Stress and anxiety: Emotional stress and anxiety can exacerbate atopic dermatitis symptoms, and some people with atopic dermatitis may also experience anxiety or depression. |
|  | Other skin infections: People with atopic dermatitis may be more prone to developing other skin infections such as impetigo, a bacterial skin infection that causes red sores on the face and other parts of the body. |
|  | It's important to note that not everyone with atopic dermatitis will develop these associated conditions, and having one or more of these conditions does not necessarily mean that a person has atopic dermatitis. It's always best to consult with a healthcare professional for an accurate diagnosis and appropriate treatment. |

The table demonstrates a sample of ChatGPT responses (with scores ranging from 1–3) for potential patient questions for atopic dermatitis and acne vulgaris.
